# Quantifying bee assemblages and attractiveness of flowering woody landscape plants for urban pollinator conservation

**DOI:** 10.1371/journal.pone.0208428

**Published:** 2018-12-26

**Authors:** Bernadette M. Mach, Daniel A. Potter

**Affiliations:** Department of Entomology, University of Kentucky, Lexington, Kentucky, United States of America; Public Library of Science, UNITED KINGDOM

## Abstract

Urban and suburban landscapes can be refuges for biodiversity of bees and other pollinators. Public awareness of declining pollinator populations has increased interest in growing plants that provide floral resources for bees. Various publications and websites list “bee-friendly” plants, but such lists are rarely based on empirical data, nor do they emphasize flowering trees and shrubs, which are a major component of urban landscapes. We quantified bee visitation to 72 species of flowering woody landscape plants across 373 urban and suburban sites in Kentucky and southern Ohio, USA, sampling and identifying the bee assemblages associated with 45 of the most bee-attractive species. We found strong plant species effects and variation in seasonal activity of particular bee taxa, but no overall differences in extent of bee visitation or bee genus diversity between native and non–native species, trees and shrubs, or early-, mid-, and late-season blooming plants. Horticulturally-modified varieties of *Hydrangea*, *Prunus*, and *Rosa* with double petals or clusters of showy sterile sepals attracted few bees compared to related plants with more accessible floral rewards. Some of the non-native woody plant species bloomed when floral resources from native plants were scarce and were highly bee-attractive, so their use in landscapes could help extend the flowering season for bees. These data will help city foresters, landscape managers, and the public make informed decisions to create bee–friendly urban and suburban landscapes.

## Introduction

Many wild bee species, including important crop pollinators such as bumble bees (*Bombus* spp.), are declining in abundance or range [1–6]. Loss of floral resources, associated with agricultural intensification and habitat loss, is one of the major drivers of pollinator decline [5,7]. Protecting natural areas and restoring agricultural lands are important strategies for pollinator conservation, but urban landscapes, which offer a variety of forage and nesting sites, can also be refuges for bees [8–10]. Indeed, substantial portions of native bee communities can persist and even thrive in urban and suburban areas with support from gardens [11–16], parks [17], low-input lawns [18–19], and other properly designed and managed urban green spaces.

Bees are keystone species in urban environments, where their pollination services help propagate both wild and ornamental plants that in turn support birds and other urban wildlife by providing fruit and seeds as well as harboring insect prey [1,20–22]. Urban bees directly benefit people by pollinating crops grown in residential and community gardens [23,24], but they also present opportunities to interact with nature and engage in conservation [25–28]. The rise in urban honey beekeeping [29] and initiatives such as the "Million Pollinator Garden Challenge" [30], the Monarch Waystation program [31], and the Certified Wildlife Habitat program [32] in the United States, and the Royal Horticultural Society's "Plants for Pollinators" [33] and Buglifes "B-lines" network of wildflower-rich habitat [34] in Great Britain have spurred public interest and participation in gardening or landscaping to help conserve pollinators, and many garden centers and websites now promote certain species or varieties of ornamental plants as "friendly" to bees, butterflies, and other flower-visiting insects [35,36].

Numerous lists of "pollinator friendly" plants have been compiled by conservation organizations [33, 37–38], or produced by individuals and published in books [39,40] or on websites. Those lists, for the most part, are not well-grounded in empirical data [35] or do not cite published sources of such data, nor do they specify, except in general terms (e.g. "bees", "butterflies", or "flies"), the taxonomic composition of pollinator assemblages attracted to particular plant species. With > 4000 species of native bees in North America [41], each with unique life history and feeding preferences, such lists have limited conservation value. Another shortcoming is that, unlike the Royal Horticultural Society's compilation of pollinator-attractive garden plants for Great Britain [33] which includes both herbaceous and woody plants, existing lists from North American invertebrate conservation organizations focus mainly on native herbaceous plants. For example, for the region of the United States that includes Kentucky, Pollinator Partnership's planting guide lists only 13 species of bee-attractive trees and shrubs, and Xerces Society's list of "Pollinator Plants" (Southeast Region) includes only seven [37,38]. Several scientific studies have documented the genera or species of bees associated with native eastern North American herbaceous perennials [42] and selected herbaceous native and non–native garden plants [12,16,36,43,44], but no comparable studies have documented the bee assemblages associated with a broad array of woody landscape plants anywhere in North America.

Flowering woody plants can provide valuable food resources for urban bee populations [22,45]. A single tree or large shrub can produce thousands of flowers, far more per unit area than in an equivalent patch of garden plants or meadow, and offer copious pollen and nectar with high sugar content [45]. Landscapes with a mix of woody plants whose collective bloom periods extend from early spring to autumn can buffer bee populations from seasonal gaps in floral resource availability that can occur with herbaceous ornamental flowers in urban gardens [12,46]. Such landscapes also promote bee species richness and diversity by sustaining early–emerging seasonal specialists (e.g., *Andrena* spp.) as well as eusocial species (e.g., honey bees and bumble bees) whose colony development and reproduction requires large amounts of pollen and nectar throughout the growing season [45,47]. Establishing sustainable woody landscape plants to provide more and better food for bees should be part of any strategy to conserve and restore urban pollinators.

About 75% of all U.S. households engage in yard and garden activities [48], so there is a need for actionable science to help city foresters, landscapers, and a larger, interested public make informed decisions in creating bee–friendly landscapes. To that end, we quantified bee visitation to a wide range of established flowering trees and shrubs at 373 urban and suburban sites in central and northern Kentucky and southern Ohio, USA, and sampled the bee assemblages associated with 45 of the most bee–attractive plant species. Although wind-pollinated plants can serve as important pollen sources for spring-active bees [49–51], we focused on insect-pollinated trees and shrubs that are attractive to consumers in part because of their showy flowers or fruits. We compared overall attractiveness and bee genus richness and diversity between native and non-native plant species, trees and shrubs, and early-, mid-, and late-season blooming species. Patterns of preference and seasonal activity of different bee taxa based on their abundance in collections from each plant species were quantified. We identified numerous bee-attractive species of woody landscape plants and documented clear differences in the assemblages of bees attracted to different plant species.

## Materials and methods

### Plant species

In total, 72 species of flowering woody plants were sampled from 2014–2017 (Tables 1 and 2). Sampling took place from February to November each year. Plant species were selected based on recommendations from land care professionals, their suitability for planting within the Ohio River Valley region, and availability and frequency of use in urban landscapes. Both native and non-native plant species were included in order to compare their usage by bees. Plants listed as an invasive or nuisance species by the USDA National Invasive Species Information Center [52] or by the state governments of Kentucky, Tennessee, Missouri, Illinois, Indiana, Ohio, West Virginia, or Virginia were not included. Additionally, we sampled three sets of plant species (*Hydrangea* spp., *Ilex* spp., and *Rosa* spp.) to compare bee–attractiveness and bee genus diversity among cultivars differing in horticultural characteristics, and between closely-related native and non-native plants.

**Table 1:** Plant characteristics and snapshot counts of 36 flowering trees. Snapshot counts are presented as mean (range).

Species	Common Name	n	Plant Family	Flower Color^a^	Flower Type	Inflorescence Type	Prov^b^	Bloom Period
*Aesculus flava*	Yellow buckeye	3	Sapindaceae	Y	tubular	raceme	nat	Apr–May
*Aesculus* × *carnea*	Red horsechestnut	5	Sapindaceae	PK	tubular	raceme	non	Apr–May
*Amelanchier* spp.	Serviceberry	4	Rosaceae	W	rosaceous	raceme	nat	Apr–May
*Aralia elata*, *spinosa*^c^	Devil's walking stick	5	Araliaceae	W	cruciate	raceme	varies	Jul–Aug
*Catalpa speciosa*	Catalpa	3	Bignoniaceae	W	funnel	panicle	nat	May–Jun
*Cercis canadensis*	Eastern redbud	5	Fabaceae	PP	papilionaceous	umbel	nat	Apr–May
*Chionanthus virginicus*	White fringetree	5	Sapindaceae	W	cruciate	umbel	nat	May–Jun
*Cladrastis kentukea*	American yellowwood	5	Fabaceae	W	papilionaceous	raceme	nat	May–Jun
*Cornus drummondii*	Roughleaf dogwood	5	Cornaceae	W	cruciate	umbel	non	May–Jun
*Cornus florida*	Flowering dogwood	5	Cornaceae	W	cruciate	umbel	nat	Apr–May
*Cornus kousa*	Kousa dogwood	5	Cornaceae	W	cruciate	umbel	non	May–Jun
*Cornus mas*	Cornelian cherry	5	Cornaceae	Y	cruciate	umbel	non	Mar
*Crateagus viridus*	Winter king hawthorn	5	Rosaceae	W	rosaceous	corymb	nat	Apr–May
*Heptacodium miconioides*	Seven–son flower	5	Caprifoliaceae	W	cruciate	umbel	non	Aug–Sep
*Ilex opaca*	American holly	5	Aquifoliaceae	W	cruciate	umbel	nat	Apr–May
*Ilex* × *meserveae*	Blue/China holly	5	Aquifoliaceae	W	cruciate	umbel	non	Apr–May
*Koelreuteria paniculata*	Golden raintree	5	Sapindaceae	Y	rotate	panicle	non	Jun–Jul
*Lagerstroemia* sp.	Crape myrtle	4	Lythraceae	PK	cruciate	raceme	non	Jul–Aug
*Maackia amurensis*	Amur maackia	3	Fabaceae	W	papilionaceous	spike	non	Jul
*Magnolia liliiflora*	Mulan magnolia	5	Magnoliaceae	PP	cup	raceme	non	Apr–May
*Magnolia stellata*	Star magnolia	5	Magnoliaceae	W	cup	raceme	non	Mar
*Malus* spp.	Flowering crabapple	5	Rosaceae	W	rosaceous	corymb	varies	Mar–Apr
*Nyssa sylvatica*	Black gum, Tupelo	5	Cornaceae	W	cruciate	umbel	nat	Apr–Jun
*Oxydendrum arboreum*	Sourwood	5	Ericaceae	W	urceolate	raceme	nat	Jun–Jul
*Prunus* 'kanzan'	Japanese cherry	5	Rosaceae	PK	rosaceous	umbel	non	Apr
*Prunus* spp.	Flowering cherry	4	Rosaceae	PK	rosaceous	umbel	varies	Mar–Apr
*Prunus subhirtella* 'Autumnalis'	Higan cherry	4	Rosaceae	PK	rosaceous	umbel	non	Mar–Apr
*Prunus subhirtella* 'Pendula'	Higan weeping cherry	5	Rosaceae	PK	rosaceous	umbel	non	Mar–Apr
*Prunus virginiana*	Chokecherry	4	Rosaceae	W	rosaceous	panicle	nat	Apr–May
*Rhus copallinum*	Winged sumac	5	Anacardiaceae	W	rotate	panicle	nat	Jul–Aug
*Sambucus canadensis*	American elderberry	5	Adoxaceae	W	rotate	cyme	nat	Jun
*Sassafras albidum*	Sassafras	3	Lauraceae	Y	rotate	umbel	nat	Apr
*Syringa reticulata*	Japanese Tree Lilac	5	Oleaceae	W	cruciate	panicle	non	May–Jun
*Tetradium daniellii*	Bee bee tree	4	Rutaceae	W	rotate	umbel	non	Jul–Aug
*Tilia cordata*	Linden	5	Tiliaceae	W	rotate	umbel	non	Jun–Jul
*Vitex agnus-castus*	Chaste tree	5	Lamiaceae	W	funnel	umbel	non	Jul–Aug

^a^W = white, Y = yellow, R = red, Pk = pink, Pp = purple, G = green, B = blue

^b^Prov = provenance; nat = native; non = non–native; varies = includes both native and non–native species

^c^*Aralia elata* (non) and *A*. *spinosa* (nat) are closely-related and cannot be reliably distinguished from one another in the field

**Table 2:** Plant characteristics and snapshot counts of 36 flowering shrubs. Snapshot counts are presented as mean (range).

Species	Common Name	n	Plant Family	Flower Color^a^	Flower Type	Inflorescence Type	Prov^b^	Bloom Period
*Abelia* × *grandiflora*	Abelia	5	Caprifoliaceae	PK	funnel	cyme	non	Jul–Sep
*Aesculus parviflora*	Bottlebrush buckeye	5	Sapindaceae	W	tubular	raceme	nat	Jun–Jul
*Amorpha fruticosa*	False indigo	5	Sapindaceae	PP	tubular	raceme	nat	May–Jun
*Buxus sempervirens*	European boxwood	5	Buxaceae	G	apetalous	panicle	non	Mar–Apr
*Calycanthus floridus*	Carolina allspice	3	Calycanthaceae	R	rosaceous	panicle	nat	Apr–May
*Cephalanthus occidentalis*	Buttonbush	4	Rubiaceae	W	tubular	head	nat	Jun
*Clethra alnifolia* '16 candles'	Summersweet	5	Clethraceae	W	rotate	spike	nat	Jul–Aug
*Deutzia scabra*	Fuzzy deutzia	5	Hydrangeaceae	W	cruciate	corymb	non	May
*Forsythia* spp.	Forsythia	5	Oleaceae	Y	cruciate	umbel	non	Mar
*Fothergilla gardenii*	Dwarf fothergilla	5	Hamamelidaceae	W	apetalous	spike	nat	Mar–Apr
*Hamamelis vernalis*	Ozark witch hazel	4	Hamamelidaceae	Y	cruciate	raceme	nat	Jan–Mar
*Hydrangea arborescens* ‘Annabelle’	Smooth hydrangea ‘Annabelle’	5	Hydrangeaceae	W	cruciate	corymb	nat	Jun
*Hydrangea macrophylla*	Bigleaf hydrangea	5	Hydrangeaceae	B	cruciate	corymb	non	Jun
*Hydrangea paniculata*	PG Hydrangea	5	Hydrangeaceae	W	cruciate	panicle	non	Jul–Aug
*Hydrangea quercifolia*	Oakleaf hydrangea	5	Hydrangeaceae	W	cruciate	panicle	nat	Jun–Jul
*Hypericum frondosum*	St.John's wort	5	Hypericaceae	Y	rosaceous	raceme	nat	Jun–Jul
*Hypericum* 'Hidcote'	St. John's wort 'Hidcote'	4	Hypericaceae	Y	rosaceous	raceme	non	Jun
*Ilex verticillata*	Common winterberry	5	Aquifoliaceae	W	cruciate	umbel	nat	Jun
*Ilex* × *attenuata*	Foster's holly	5	Aquifoliaceae	W	cruciate	umbel	nat	Apr–May
*Itea virginica*	Sweetspire	5	Iteaceae	W	rotate	raceme	nat	Jun–Jul
*Lindera benzoin*	Spicebush	5	Lauraceae	Y	rotate	panicle	nat	Mar
*Lonicera fragrantissima*	Winter honeysuckle	5	Caprifoliaceae	W	funnel	umbel	non	Mar–Apr
*Philadelphu*s spp.	Mock orange	5	Hydrangeaceae	W	cruciate	raceme	varies	May–Jun
*Physocarpus opulifolius*	Ninebark	3	Rosaceae	W	rosaceous	corymb	nat	May–Jun
*Prunus laurocerasus*	Cherry laurel	5	Rosaceae	W	rosaceous	spike	non	Apr–May
*Pyracantha* spp.	Pyracantha	4	Rosaceae	W	rosaceous	corymb	non	May
*Rhododendron* spp.	Azalea	5	Ericaceae	W	rosaceous	umbel	non	Apr–May
*Rhododendron* spp.	PJM rhododendron	5	Ericaceae	PK	rosaceous	umbel	non	Mar–Apr
*Rosa setigera*	Climbing rose	5	Rosaceae	PK	rosaceous	head	nat	Jun
*Rosa* spp.	Hybrid tea rose	35	Rosaceae	PK	rosaceous	head	non	May–Oct
*Spiraea* × *vanhouttei*	Vanhoutte spiraea	5	Rosaceae	W	rosaceous	umbel	non	Apr–May
*Spirea japonica*	Japanese spirea	5	Rosaceae	PK	rosaceous	corymb	non	May–Jun
*Spirea virginiana*	Virginia spiraea	5	Rosaceae	W	rosaceous	corymb	nat	May–Jun
*Syringa vulgaris*	Lilac	5	Oleaceae	PP	cruciate	panicle	non	Apr–May
*Viburnum burkwoodii*	Burkwood viburnum	5	Adoxaceae	W	funnel	cyme	non	Apr
*Viburnum carlesii*	Koreanspice viburnum	5	Adoxaceae	W	funnel	cyme	non	Apr–May

^a^W = white, Y = yellow, R = red, Pk = pink, Pp = purple, G = green, B = blue

^b^Prov = provenance; nat = native; non = non–native; varies = includes both native and non–native species

### Sample sites

All 373 sample sites were located within the urban landscape and were separated by at least 1 km for same–species sites to ensure minimal overlap of bee populations. Sample sites included street-side and municipal plantings, commercial and residential landscapes, campuses, parking lots, and urban arboreta and cemeteries. Most (93%) of the sample sites were within the Lexington, Kentucky USA metropolitan area; the remainder were in urban or peri-urban cemeteries or arboreta in Louisville or northern Kentucky, or near Cincinnati in southern Ohio. All sample sites were within 145 km of the Lexington city limits. Individual sample sites ranged from single trees or large shrubs, to groupings or hedges of a particular plant species. We sampled five different sites for most (56) of the 72 plant species, four sites for 10 plant species, and three sites for each of the remaining six, harder-to-locate woody plants. An additional 35 sites were used for comparisons between native and hybrid tea roses (*Rosa* spp.). Permissions to collect samples of bees were granted by grounds managers, staff, or by property owners depending on site type.

### Bee–attractiveness ratings

Given the wide variation in plant height and form, and in floral density, size, and morphology across such a wide range of trees and shrubs planted at hundreds of sites, it was not possible to standardize sampling on the basis of floral area such as has been done in studies [e.g., 15,40] quantifying bee visitation to same-sized replicated plots of herbaceous flowering plants in a common-garden setting. Instead, each plant species’ relative bee–attractiveness was rated based on two 30-second “snapshot” counts [16] per site for in most cases 10 (minimum of six) snapshot counts per plant species. The snapshot counts were also used to justify the exclusion of relatively non-attractive plants from more extensive bee sampling. Snapshot counts were taken at or near peak bloom of a given plant. During each 30-second period, bees actively foraging on the flowers of the target plant(s) were counted, taking care to avoid counting the same insects more than once. Snapshot counts were taken while walking slowly around the tree or shrub, or along hedges or other sites with long, continuous plantings (> 2.5 m), whereas for smaller shrubs they were taken while stationary. For relatively tall trees, snapshot counts were taken only as high up in the canopy as the observer was able to distinguish bees from flies or other insects. Because of the large number of sample sites and distances between them, variable weather conditions, and the relatively brief (1–2 week) and overlapping bloom periods for many of the plants, in most cases it was not possible to visit and sample a given site more than once. Sampling conditions were as consistent as possible within a given species; e.g., same-species sites were sampled on the same day or within a few days of each other in a given year, snapshot counts were taken between 10:00 to 18:00 EST during non-inclement weather (e.g., sunny to partly cloudy, winds < 16 kph), and sampling of early spring-blooming species was done only on days with temperatures >10°C and bees were active. Snapshot counts at a given site were taken immediately before collecting each 50-bee sample (see below) to minimize disturbance of the bees.

### Sample collection

We sampled the bee assemblages associated with 45 of the 72 aforementioned plant species, excluding relatively non–attractive ones with average snapshot counts of < 5 bees. Samples were collected from 213 total sites including five sites for 35 of the plant species, four sites for eight species, and three sites for two of the rarer plants (213 total sites). Bees were collected immediately after taking snapshot counts and represented the first 50 bees observed on the flowers after the counts were finished (250 total bees collected for most plant species). Most samples were collected using aerial insect nets that could be extended to collect from heights up to about 5 m above ground level, when necessary. Some shrubs with fragile flowers were sampled by knocking individual bees into plastic containers filled with 75% EtOH. Sampling time ranged from < 15 min to more than 2 h per site. Bee samples were washed with water and dish soap, rinsed, then dried using a fan–powered dryer for 30–60 min, and pinned. All bees were identified to genus [53,54]. Bumble bees (*Bombus* spp.) and honey bees (*Apis mellifera* L.) were identified to species. Reference specimens are deposited in the University of Kentucky Department of Entomology Insect Collection.

### Statistical analysis

Snapshot counts, bee genus diversity, and abundances of each of the five predominant families of bees (Andrenidae, Apidae, Colletidae, Halictidae, Megachilidae) and of *Bombus* spp. and *A*. *mellifera*, were compiled across sampling years and analyzed for main effects of plant species, plant family (as a proxy for plant species due to limited degrees of freedom), provenance (native or non-native), plant type (tree or shrub), and Julian date number for peak bloom using General linear models procedure (SAS, Version 9.4; SAS Institute, Cary, NC, USA). Species diversity was based on the inverse of Simpson’s D (hereafter 1/D), which calculates a number between 0 and 1, with higher numbers indicating more species–rich and even samples (Margarun 2004). For analysis of bee taxa abundance, we counted the number of individuals in each sample belonging to one of five North American bee families (Apidae, Andrenidae, Colletidae, Halictidae, Megachilidae) and two additional taxa, *Apis mellifera* and *Bombus* spp. and analyzed abundance of each for main effects. Sampling date was standardized by converting to a Julian date number, which assigns each calendar date a unique integer starting from 0 on January 1. We also attempted to analyze main effects of flower color, flower type, and inflorescence type on bee snapshot counts, taxa abundance, and diversity but were unable to do so due to the uneven distribution of the data among class variable levels.

## Results

### Bee–attractiveness ratings

**Fig 1:**
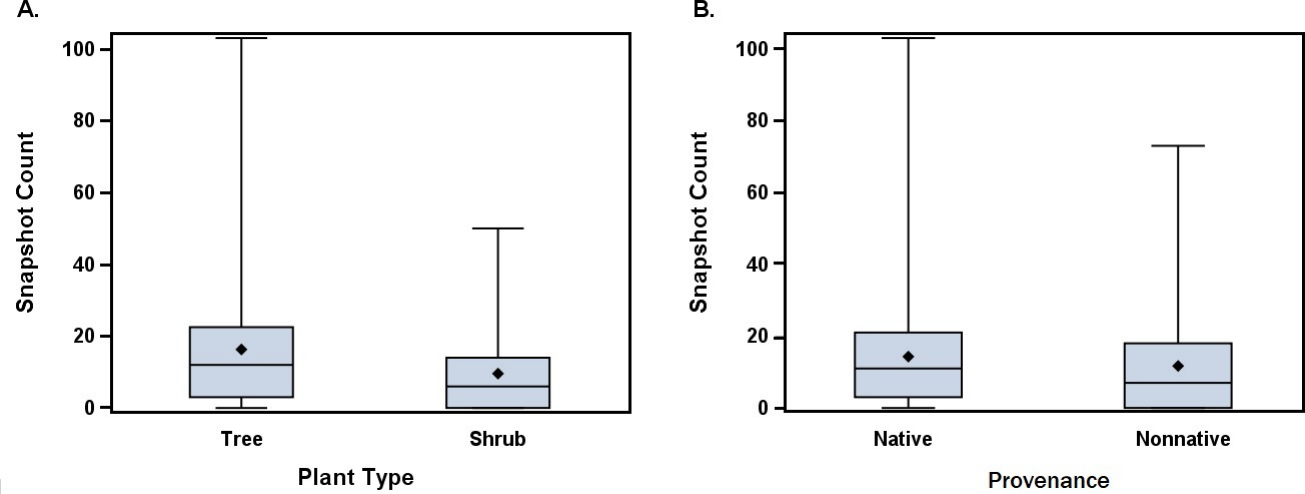
Comparison of snapshot counts on trees and shrubs, and native and non–native plants. The bold line within the box indicates the median while the diamond indicates the mean. The lower whisker, lower box, upper box, and upper whisker indicate first, second, third, and fourth quartiles, respectively. Whiskers also show minimum and maximum values (range). Analysis of variance results are summarized in Table 4.

Snapshot counts, which were obtained for all 72 plant species, ranged from 0 to 103 with an average count of 12.8 bees per 30-second observation per site. Plants' general attractiveness ratings are summarized in Table 3. The plants with the five highest average snapshot counts were *Rhus copallinum*, *Tetradium daniellii*, *Maackia amurensis*, *Heptacodium miconioides*, and *Hydrangea paniculata* (65.3, 50.1, 42.2, 33.2, and 31.4, respectively). We did not observe any bees during the snapshot counts for *Calcycanthus floridus*, *Hydrangea arborescens* ‘Annabelle’, *Hydrangea macrophylla*, *Magnolia liliiflora*, and *Sassafras albidum* at any of the sites sampled. Plant species and family, plant type (tree or shrub), and Julian date number had significant effects on snapshot counts (Table 4). There were small but statistically significant differences in snapshot counts between trees and shrubs, with trees having higher snapshot counts than shrubs (Fig 1). Snapshot counts increased slightly as the growing season progressed. There were no significant differences in snapshot counts between native or non-native species (Fig 1).

**Table 3:** Bee-attractiveness rating^a^, distribution of bee taxa^b^, bee genus diversity^c^, and provenance^d^ of 45 species of bee-attractive flowering trees and shrubs. Plant species are arranged in order of bloom period.

Species	Sites	Bees (n)	Bloom Period	Prov^d^	Rating	Apid	*A*. *mellifera*	*Bombus* spp.	Andr	Coll	Hali	Mega	Diversity
*Cornus mas*	5	247	Mar	non	***	45.7	45.7	0	43.7	2.4	6.5	1.6	0.34
*Fothergilla gardenii*	5	267	Mar–Apr	nat	*	18.0	1.1	0.7	70.0	1.9	9.7	0.4	0.43
*Malus* spp.	5	258	Mar–Apr	varies	****	10.1	7.4	1.2	65.1	2.7	16.3	5.8	0.47
*Prunus* spp.	4	194	Mar–Apr	varies	****	19.1	11.9	0.5	66.0	4.1	3.6	7.2	0.49
*Prunus subhirtella* 'Autumnalis'	4	213	Mar–Apr	non	***	84.5	83.1	0.9	3.8	2.8	2.8	6.1	0.27
*Prunus subhirtella* 'Pendula'	5	285	Mar–Apr	non	**	22.5	18.6	1.8	18.2	39.6	6.3	13.3	0.62
*Viburnum burkwoodii*	5	284	Apr	non	**	13.7	9.5	1.4	18.3	1.1	46.8	20.1	0.58
*Aesculus* × *carnea*	5	282	Apr–May	non	*	63.1	40.8	14.2	28.7	2.8	1.8	3.5	0.70
*Amelanchier* spp.	4	215	Apr–May	nat	***	4.7	2.8	0	80.5	0.9	14.0	0	0.23
*Cercis canadensis*	5	274	Apr–May	nat	****	8.0	0	1.1	1.5	79.2	2.2	9.1	0.31
*Cornus florida*	5	155	Apr–May	nat	*	4.5	2.6	0.6	79.4	11.6	4.5	0	0.35
*Crateagus viridus*	5	345	Apr–May	nat	**	21.7	15.1	2.3	51.3	4.6	22.0	0.3	0.57
*Ilex opaca*	5	242	Apr–May	nat	**	60.3	21.1	2.1	30.2	0.4	8.3	0.8	0.65
*Ilex* × *attenuata*	5	302	Apr–May	nat	**	65.6	46.0	3.3	5.6	3.3	23.8	1.7	0.51
*Ilex* × *meserveae*	5	254	Apr–May	non	**	51.2	39.4	0	16.9	9.1	17.7	5.1	0.60
*Nyssa sylvatica*	5	268	Apr–May	nat	**	28.0	21.3	3.4	42.9	4.9	23.9	0.4	0.32
*Prunus laurocerasus*	5	273	Apr–May	non	*	4.4	0.7	1.1	61.5	0	33.3	0.7	0.47
*Prunus virginiana*	4	220	Apr–May	nat	***	4.1	1.8	0	76.8	1.8	17.3	0	0.35
*Deutzia scabra*	5	245	May	non	*	65.3	14.7	8.6	27.3	1.2	4.1	2.0	0.57
*Pyracantha* spp.	4	238	May	non	*	8.4	1.3	3.8	69.7	4.2	17.2	0.4	0.47
*Amorpha fruticosa*	5	302	May–Jun	nat	**	76.8	25.5	27.8	10.9	1.0	7.6	3.6	0.66
*Cladrastis kentukea*	5	268	May–Jun	nat	*	88.8	67.9	14.9	9.7	0	1.1	0.4	0.48
*Philadelphu*s spp.	5	253	May–Jun	varies	**	5.9	0.8	3.6	1.2	0.8	5.5	86.6	0.24
*Physocarpus opulifolius*	3	167	May–Jun	nat	**	20.4	7.8	1.2	57.5	6.0	16.2	0	0.60
*Spirea virginiana*	5	277	May–Jun	nat	***	33.9	0.7	12.3	49.5	6.1	10.5	0	0.67
*Syringa reticulata*	5	221	May–Jun	non	*	31.2	7.2	3.6	48.0	0.5	20.4	0	0.64
*Cephalanthus occidentalis*	4	199	Jun	nat	**	48.7	3.5	45.2	0	0.5	49.7	1.0	0.50
*Ilex verticillata*	5	267	Jun	nat	****	59.6	54.3	2.6	2.2	1.5	19.1	17.6	0.53
*Rosa setigera*	5	160	Jun	nat	**	59.4	16.3	41.3	0.6	6.3	31.3	2.5	0.70
*Aesculus parviflora*	5	260	Jun–Jul	nat	***	64.2	25.8	8.1	0.4	1.2	32.3	1.9	0.71
*Hypericum frondosum*	5	268	Jun–Jul	nat	****	70.1	33.6	35.8	0	1.9	28.0	0	0.51
*Itea virginica*	5	270	Jun–Jul	nat	*	80.4	19.6	17.0	9.6	4.1	3.7	2.2	0.65
*Koelreuteria paniculata*	5	282	Jun–Jul	non	***	57.8	42.6	9.6	1.1	0	39.0	2.1	0.59
*Oxydendrum arboreum*	5	228	Jun–Jul	nat	***	59.6	4.4	51.8	0	0.4	31.1	8.8	0.69
*Tilia cordata*	5	264	Jun–Jul	non	***	80.7	48.1	15.9	1.5	0	17.4	0.4	0.60
*Maackia amurensis*	3	165	Jul	non	****	26.7	6.7	12.1	0	0	44.8	28.5	0.63
*Aralia elata*, *spinosa*	5	270	Jul–Aug	varies	***	26.3	22.2	1.1	0	4.1	68.5	1.1	0.34
*Clethra alnifolia* '16 candles'	5	260	Jul–Aug	nat	****	48.5	2.7	39.6	0	2.7	46.2	2.7	0.52
*Hydrangea paniculata*	5	283	Jul–Aug	non	****	26.5	25.1	0.7	0.4	0.7	72.4	0	0.42
*Lagerstroemia* sp.	4	220	Jul–Aug	non	*	37.3	26.4	5.0	0	0.9	61.8	0	0.41
*Rhus copallinum*	5	269	Jul–Aug	nat	****	68.0	60.6	6.3	0	0.7	31.2	0	0.41
*Tetradium daniellii*	4	258	Jul–Aug	non	****	70.5	64.7	5.0	0.4	0.8	19.8	8.5	0.44
*Vitex agnus-castus*	5	263	Jul–Aug	non	*	61.6	2.3	43.7	0	0.8	22.1	15.6	0.64
*Abelia* × *grandiflora*	5	275	Jul–Sep	non	***	48.4	2.9	31.6	0	0	44.0	7.6	0.74
*Heptacodium miconioides*	5	265	Aug–Sep	non	****	72.1	12.8	49.1	0	1.1	26.4	0.4	0.52

^a^Bee–attractiveness ratings are based on quartiles of snapshot counts, with * = first quartile, ** = second quartile, *** = third quartile, and **** = fourth quartile

^b^Bee taxa distribution is presented as percentage of total bees collected for each plant species. Andr = Andrenidae, Coll = Colletidae, Hali = Halictidae, Mega = Megachilidae

^c^Diversity is calculated as the inverse of Simpson's D, which generates a number between 0 and 1 with higher values indicating more genus–rich and even samples

^d^Prov = provenance; nat = native; non = non-native; varies = included both native and non-native species

**Table 4:** Summary of analysis of variance for effects of plant species, plant type (tree or shrub), provenance (native or non-native), and Julian date number on bee genus diversity and snapshot counts.

	Snapshot counts			Bee genus diversity		
Source	df	*F*	Pr >*F*	df	*F*	Pr >*F*
Plant species	70	18.40	<0.001	42	2.60	<0.001
Plant family	25	13.97	<0.001	20	2.60	<0.001
Plant type	1	20.37	<0.001	1	0.68	0.41
Provenance	1	1.28	0.26	1	1.11	0.29
Julian date number	1	10.63	<0.001	1	3.60	0.06

### Bee abundance by taxon and genus diversity

Overall, 11,275 bees were collected from 45 species of flowering woody plants that attracted, on average, ≥ 5 bees in the snapshot counts. Apid bees comprised 44.0% of all bees sampled and were present on all 45 plant species sampled (Tables 3 and 5). Halictid bees were similarly ubiquitous on all plant species and accounted for 23.6% of total bees. Andrenid bees accounted for 21.4% of total bees and often dominated the bee assemblages of early blooming plants. Colletid and megachilid bees were the least abundant bees overall, comprising only 5.0 and 5.9%, respectively, of the total bees in our samples. *Apis mellifera* and *Bombus* spp. were collected from 44 and 39 of the sampled plant species, respectively, and accounted for 21.4 and 11.9% of the total bees.

**Table 5:** Characteristics and distribution of bee species and genera identified on 45 species of flowering woody plants.

Family	Genus/Species	Nesting Habit	Nest Type	% of Family	% of Total
Apidae	*Apis mellifera*	social	cavity	50.1	22.1
	*Bombus auricomus*	social	above–ground	< 0.1	< 0.1
	*Bombus bimaculatus*	social	ground/cavity	4.3	1.9
	*Bombus citrinus*	solitary	kleptoparasitic	< 0.1	< 0.1
	*Bombus griseocolus*	social	ground	7.7	3.4
	*Bombus impatiens*	social	ground	15.8	6.9
	*Bombus pennsylvanicus*	social	above–ground	< 0.1	< 0.1
	*Bombus perplexus*	social	ground	< 0.1	< 0.1
	*Ceratina*	varies	cavity	5.0	2.2
	*Melissodes*	solitary	ground	0.1	< 0.1
	*Nomadinae*	solitary	kleptoparasitic	0.8	0.4
	*Xylocopa*	varies	cavity	16.0	7.1
Andrenidae	*Andrena*	solitary	ground	100.0	21.4
Colletidae	*Colletes*	solitary	ground	75.3	3.8
	*Hylaeus*	solitary	cavity	24.7	1.2
Halictidae	*Agapostemon*	solitary	ground	1.6	0.4
	*Augochlora*	solitary	cavity	8.5	2.0
	*Augochlorella*	solitary	ground	1.4	0.3
	*Augochloropsis*	solitary	ground	1.2	0.3
	*Halictus*	solitary	ground	3.8	0.9
	*Lasioglossum*	varies	ground	83.0	19.6
	*Sphecodes*	solitary	kleptoparasitic	0.5	0.1
Megachilidae	*Anthidium*	solitary	cavity	0.1	< 0.1
	*Chelostoma*	solitary	cavity	32.4	1.9
	*Coelioxys*	solitary	kleptoparasitic	0.4	< 0.1
	*Heriades*	solitary	cavity	8.0	0.5
	*Hoplitis*	solitary	cavity	0.3	< 0.1
	*Megachile*	solitary	cavity	29.8	1.8
	*Osmia*	solitary	cavity	28.9	1.7

Plant species (Table 4), and by extension plant family, played a key role in abundance of all bee taxa analyzed (Table 6) and both were the only significant factors for Andrenidae, Apidae, and *A*. *mellifera*. Most woody plants attracted bees from at least four families; one exception was mock orange (*Philadelphus*) from which >95% of the bees collected were *Chelostoma philadelphi* (Robertson), a small megachilid. Colletidae, Halictidae, and *Bombus* all showed strong seasonal patterns in abundance, with the proportion of Colletidae in our samples declining sharply with increasing Julian date, while proportionate abundance of Halictidae and *Bombus* increased. Colletidae were proportionately more abundant on trees than on shrubs, and on native as opposed to non-native plant species (Table 6). All other bee taxa, including non-native *A*. *mellifera* and native *Bombus*, were equally proportionately abundant on native and non-native plants.

**Table 6:** Summary of analysis of variance for effects of plant species, plant type (tree or shrub), provenance (native or non-native), and Julian date number on bee taxa abundance.

	Andrenidae			Apidae		Colletidae		Halictidae	
Source	df	*F*	Pr >*F*	*F*	Pr >*F*	*F*	Pr >*F*	*F*	Pr >*F*
Plant family	20	5.74	<0.001	3.67	<0.001	1.98	0.01	2.27	0.002
Plant type	1	0.01	0.94	0.03	0.86	4.39	0.04	0.39	0.54
Provenance	1	3.39	0.07	0.01	0.91	4.58	0.03	3.29	0.07
Julian date number	1	2.29	0.13	0.06	0.8	19.65	<0.001	30.7	<0.001
	Megachilidae			*Apis mellifera*		*Bombus*			
Source	df	*F*	Pr >*F*	*F*	Pr >*F*	*F*	Pr >*F*		
Plant family	20	2.37	<0.001	4.11	<0.001	5.00	<0.001		
Plant type	1	0.85	0.36	0.5	0.48	0.40	0.53		
Provenance	1	1.13	0.29	0.91	0.34	0.01	0.95		
Julian date number	1	4.24	0.04	0.69	0.41	4.77	0.03		

**Fig 2:**
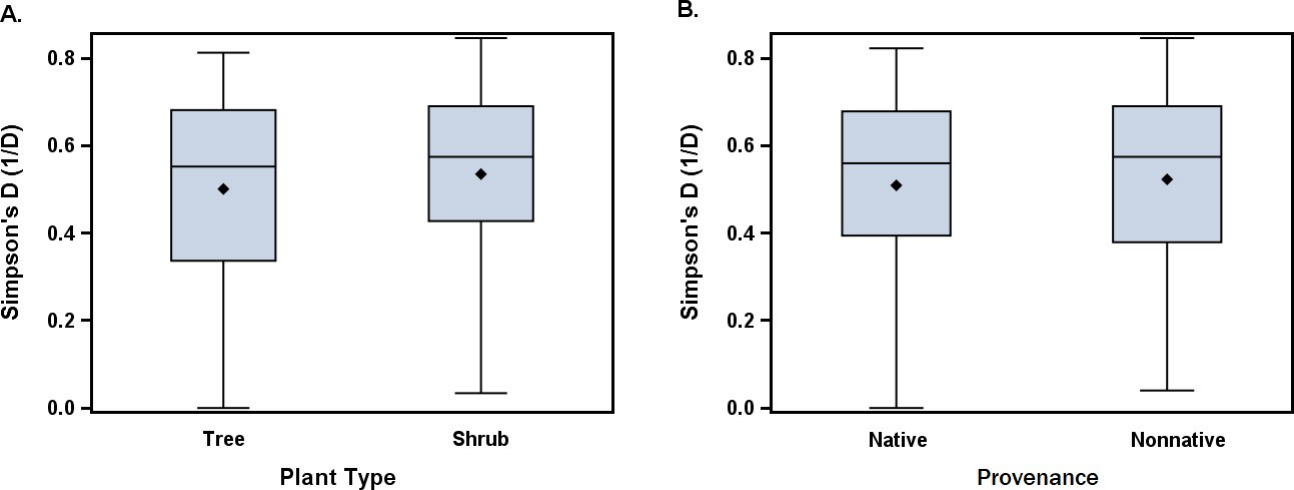
Comparison of bee genus diversity on trees and shrubs, and native and non–native plants. The bold line within the box indicates the median while the diamond indicates the mean. The lower whisker, lower box, upper box, and upper whisker indicate first, second, third, and fourth quartiles, respectively. Whiskers also show minimum and maximum values (range). Analysis of variance results are summarized in Table 4.

Twenty-three bee genera were represented in our samples (Table 5), the most abundant being *Apis* (22.1% of total bees), *Andrena* (21.4%), *Lasioglossum* (19.6%), and *Bombus* (12.2%). Bee genus diversity index values ranged from 0 to 0.85 with an average of 0.52 (Table 3). The plants with the highest average genus diversity (1/D) were *Abelia* × *grandiflora* (0.74), *Aesculus parviflora* (0.71), *Aesculus* × *carnea* (0.70), *Rosa setigera* (0.70), and *Oxydendrum arboreum* (0.69). Plant species and plant family played a key role in genus diversity (Table 4), but there were no overall significant differences in genus diversity between trees and shrubs or natives and non-natives (Fig 2).

### Cultivar comparisons

Snapshot counts were compared among four *Hydrangea* species, *H*. *arborescens* ‘Annabelle’ (native, shrub), *H*. *macrophylla* (non-native, shrub), *H*. *paniculata* (non-native, shrub), and *H*. *quercifolia* (native, shrub) which differ in their floral characteristics (Table 2). Most notably, *H*. *paniculata* has exposed fertile flowers while the other three species lack fertile flowers or have them hidden beneath showy sterile outer sepals (Dirr 2011). Non-native hydrangeas had higher average snapshot counts than native hydrangeas (14.0 and 2.8, respectively, *F*_1,34_ = 6.19, *P* = 0.02), but this was entirely because *H*. *paniculata*, a non-native, was the only species that was highly attractive to bees. Bee genus diversity was not analyzed because *H*. *arborescens*, *H*. *macrophylla*, and *H*. *quercifolia* had extremely low bee visitation rates, and were not sampled for bees.

Snapshot counts and bee assemblages were compared between four *Ilex* species: *I*. × *attenuata* (native, shrub), *I*. × *meserveae* (non-native, tree), *I*. *opaca* (native, tree), and *I*. *verticillata* (native, shrub). All four had similar floral characteristics (Tables 1 and 2) and differed mainly by height and spread of the plant. There were no significant differences between the average snapshot counts of native and non-native *Ilex* (18.8 and 15.5, respectively, *F*_1,34_ = 0.77, *P* = 0.39), nor were there significant differences between the average genus diversity of native and non-native *Ilex* species (0.56 and 0.60, respectively, *F*_1,18_ = 0.28, *P* = 0.60).

Bee visitation to two species of roses (*Rosa*) was compared. *Rosa setigera*, a single-flowered native rose with pollen prominently displayed during most of its bloom, was sampled at five sites. Hybrid tea roses are non-native, and they are typically double- or triple-flowered and either lack stamens and pollen, or have pollen that is concealed by multiple layers of petals during bloom. We sampled a variety of hybrid tea roses which we divided into seven categories based on color and flower form: light pink with single petals, dark pink with single petals, red with single petals, white with double or triple–petals, light pink with double- or triple petals, dark pink with double or triple petals, or red with double or triple–petals. *Rosa setigera* had a significantly higher average snapshot count than all hybrid tea roses sampled (16.1 and 0.1, respectively, *F*_1,78_ = 146.8, *P* < 0.001). Bee genus diversity was not analyzed because the hybrid tea roses, which had very low visitation rates, were not sampled for bees.

## Discussion

To our knowledge, this is the first scientific study to quantify variation in bee–attractiveness and bee assemblages across a wide range of flowering woody landscape plants. We identified 45 species of trees and shrubs that could be useful for augmenting floral resources for bees in urban and suburban settings. Although all of our sampling took place in Kentucky and southern Ohio, most of the bee-attractive plants on our list should grow satisfactorily throughout USDA Plant Hardiness Zone 6, which covers extensive regions of the United States [55].

As with all studies assessing diversity of bees [56], our sampling methodology has limits and biases. Counting bees on the wing, as in our snapshot counts, leaves room for misidentification (e.g., counting bee mimics as bees) and miscounting, but we attempted to reduce this by replicating counts and using skilled observers with training in bee identification. Snapshot counts and 50-bee samples were based on one visit to each site because of the large number of sample sites, the distances between sites (up to 145 km), and the relatively short bloom periods of some plants. While it is unlikely that a sample of 250 bees collected from five sites would capture the full bee species richness and diversity of a given plant species during the entirety of its bloom, our data do provide a measure of which tree and shrub species attract and support robust bee assemblages. Although some studies have used replicated plots with similar-aged plants to compare bee visitation rates [16,42], establishing 72 species of trees and shrubs in a replicated common garden plot for eventual pollinator sampling would have been impractical because of the cost, space, and time required for establishment. Moreover, results from common garden experiments can be location-specific, reflecting the relative abundance of different pollinator taxa at that particular site. Our sampling from multiple (in most cases five) plantings of each species across hundreds of existing urban landscape sites doubtless encompassed more of the variation in soil conditions, potential nesting sites, and other landscape-level factors that would affect bee diversity than if all sampling had been done at a single location.

The premise that augmenting floral resources benefits bees is based on the assumption that local bee populations are often food-limited. Floral resource availability is thought to be a major driver of population abundance and diversity of wild bees [7]. Long-term abundance of bumble bees and other wild bees has declined in parallel with widespread declines in floral abundance and diversity in Europe [1,4], and populations of solitary bees are enhanced by mass–flowering crops, suggesting that floral resources are indeed limiting [57,58]. There is some debate [59] that the dense, high-resource displays of wildflower mixes or other urban plantings intended to augment resources for bees might have unintended ecological consequences for remnant native plant biodiversity (e.g., by competing for generalist pollinators, functioning as hubs for pollinator-transmitted plant pathogens, or decreasing he likelihood of conspecific pollen transfer). However, such plantings might also increase pollination of remnant native plants through a spillover effect [59] similar to that observed in agricultural crops bordered by wildflower strips [60]. Although those types of potential ecological interactions warrant future research, they are beyond the scope of this study. Together with studies documenting that four common city tree species attracted a fifth of all native bee species occurring in Berlin, Germany[22], and that nine of the main tree species planted along streets of European cities, including some non-native species and hybrids, provide nectar and pollen of high nutritional suitability for pollinators [45] our results suggest that urban landscapes can be made even more valuable as refuges for pollinators by incorporating additional bee-attractive woody plants.

Urban and suburban landscapes typically consist of a diverse mix of native and non-native plant species [16,44,61–65]. Recently, the long–standing debate [66,67] about whether or not there is any role for non-invasive exotic plants in conservation biology has spurred a fervent movement in gardening circles advocating that urban landscapes be constructed predominantly or exclusively with native plants [68]. One of the main arguments against landscaping with non-native plants; i.e., ones that do not occur naturally in a particular region, ecosystem, or habitat, is their potential to become invasive. Although ornamental horticulture has been a major pathway for plant invasions [69,70], many non-native ornamentals are either sterile hybrids or are considered non-invasive with a low risk of escaping cultivation [71,72]. None of the woody plants included in our study is listed as invasive in Kentucky or surrounding states [52].

Another argument for landscaping with native as opposed to non-native plant species is that natives tend to support higher diversity and numbers of endemic caterpillars and other coevolved plant-feeding insects that help to sustain insectivorous birds and other desirable urban wildlife [68,73–75]. However, ornamental plants and shade trees are also valued for aesthetic appearance, so ones with abundant insect herbivores and associated feeding damage are more likely to be treated with insecticides that could be hazardous to bees. Moreover, some native North American woody plants are far more susceptible to invasive pests than their exotic congeners originating from the pest’s natal region [76,77] and thus more likely to receive pesticide applications. We identified a number of non–invasive, non–native woody plants (e.g., *Abelia*, *Aralia*, *Cornus mas*, *Heptaconium miconioides*, *Hydrangia paniculata*, *Maackia amurensis*, *Tetradium daniellii*, *Vitex agnus-castus*, and others), that are both highly bee-attractive and relatively pest free, making them good candidates for use in bee-friendly urban landscapes. The present study adds to a growing body of evidence that both native and non-native plants can be valuable in helping to support bees and other pollinators in urbanized habitats [12,16,18,22,45,46,63,64,78]. Because most urban bees are polylectic [13,14] and will forage on a wide variety of plant species, they will readily incorporate non-native plants into their diets so long as they provide sufficient quantity and quality of pollen and nectar [79].

Phenology of bloom is important when considering the value of plants for bees. Bloom time tends to be conserved by geographic origin, with cultivated non-native plants generally retaining the phenology of their source region [63,80]. Our study identified 15 species of bee-attractive woody landscape plants that typically bloom before April or after mid-July, twelve of which are non-native (Table 3). Early or late blooming plants can be especially valuable to seasonal specialists by providing floral resources during critical times of nest establishment in the spring and winter provisioning in the fall [64,81]. Bumble bees, which do not store substantial amounts of pollen and nectar, require a consistent supply of floral resources throughout the growing season including in early spring when post-wintering queens are foraging alone to establish their colony, and late in the growing season to provision the developing queen brood, and as food for new queens that feed heavily in preparation for hibernation in overwintering sites [76–78]. In our study, bumble bees constituted a large proportion of the samples from *Aesculus* × *carnea* in early spring, and from *Abelia* × *grandiflora*, *Clethra alnifolia*, *Heptacodium miconioides*, and *Vitex agnus-castus* late in the growing season. Honey bees, which will forage year-round if weather permits, also benefit from season-long floral resources. We identified a number of trees and shrubs that are highly attractive to honey bees including some that bloom early (e.g., *Cornus mas* and *Prunus subhirtella* ‘Autumnalis’) or late (e.g., *Rhus copallinum* and *Tetradium daniellii*) in the growing season. Wind-pollinated (anemophilous) plants, particularly trees, tend to flower earlier than entomophilous species [82], and they, too, can provide critical nutrients for bees, especially in early spring before the spring-summer floral resource peaks [49–51]. Inclusion of wind-pollinated trees and shrubs, which were not sampled in our study, could change the proportion of bee-attractive natives versus non-natives amongst early-blooming landscape plants. Urban landscapes can be enhanced as habitat for bees by incorporating a variety of entomophilous, anemophilous, and ambophilous flowering plant species, biased toward natives and near-natives but including some non-natives, to ensure succession of overlapping bloom periods and provide food during periods of poor nutrient availability before and after the spring to mid-summer floral resource peaks [64].

One caveat to our data suggesting that native and non-native woody landscape plants may have equivalent usefulness for urban bee conservation is the genus-level taxonomic resolution of our bee data. Without identifying all of the >11,000 bees to species, it is impossible to know whether the non-native trees and shrubs attract and support disproportionate numbers of non-native bee species. Other than the sometimes negative ecological impacts of *Apis*, *Bombus* and *Megachile* spp. that were deliberately introduced to new regions of the world for agricultural pollination [83,84], there is little or no empirical evidence that non-native bees degrade pollination networks or negatively affect the pollination of native plants [84]. However, some non-native bee species exhibit marked preferences for visiting plants from their own natal region [83] which could have consequences should those bees become invasive. In our study, the giant resin bee *Megachile sculpturalis* Smith, a native of eastern Asia, was collected from flowers of six of the 45 woody plant species from which bee assemblages were sampled, including 12 specimens collected from *Aesculus parviflora* and *Oxydendron arboreum*, and 85 specimens from *Koelreuteria paniculata*, *Tetradium daniellii*, *Vitex agnus-castus*, and *Maackia amurensis*. The first two of the aforementioned plants are native, but the latter four, which accounted for 88% of the collections, are of Asian origin. *Megachile sculpturalis* has been observed to forcibly evict and occupy the nests of native *Osmia* sp. and *Xylocopa* sp. in the United States and Europe [85], so more widespread planting of its favored Asian ornamental trees could facilitate its range expansion, with possible deleterious consequences for native bees in its introduced range. On the other hand, all of the seven *Bombus* species we collected from woody landscape plants are native, and jointly they foraged equally on native and non-native woody plants. Indeed, two of the top bumble bee "magnets" were *Abelia × grandiflora* and *Heptacodium miconioides*, both late-blooming, non-native plants that were heavily visited by *Bombus* workers and young queens into late September when most other plants in those landscapes were done blooming.

Another caveat is that, besides being used to inform decisions about woody landscape plants that may promote bee conservation, our data will be used by stakeholders wishing to identify and avoid planting trees and shrubs that attract bees, either to reduce hazard to persons having anaphylactic allergies to bee stings or general hazard and liability around residences or in public settings, or because of general fear of bees. We acknowledge that highly bee-attractive trees and shrubs that are suitable for most landscape settings might be poor choices for sites such as primary school playgrounds, yards frequented by small children, or outdoor public outdoor eating spaces.

Although some plant varieties with double flowers or showy sterile outer sepals that inhibit access to central, fertile flowers may not provide sufficient floral rewards to attract bees [46,86], many horticulturally-modified plants, including hybrids, can be as attractive, or more attractive, than their wild-type counterparts [16,44]. In our study, neither of the native *Hydrangea* species, having been bred for large clusters of showy sterile sepals, was bee-attractive whereas the open-flowered, non-native *H*. *paniculata* had the highest average snapshot count of the 36 shrub species we sampled. Similarly, *R*. *setigera*, a native single-flowered rose, was highly attractive to bees, whereas none of the double- and triple-flowered hybrid tea rose cultivars attracted more than a single bee. All four of the *Ilex* species we compared, representing a mix of native, non-native, and hybrid species, offer easily accessible floral rewards, and all four were attractive to bees. This further illustrates that cultivars and non-native species can be equally attractive to bees as long as floral rewards have not been bred out or obscured. Similar patterns were seen within other plant genera; e.g., *Prunus subhirtella* and *P*. *virginiana* that have single, open flowers, were highly bee-attractive, whereas *P*. *kanzan*, a double-flowered species, attracted almost no bees.

In conclusion, this study identified many species of flowering trees and shrubs that are highly attractive to bees and documented the types of bees that visit them. Even so, we did not come close to capturing the enormous diversity of flowering woody landscape plants available in the marketplace [62], so there is great potential for identifying additional plants that could have value for urban bee conservation. Recommendations for bee-attractive plants that are based on empirical data are preferable to the large number of plant lists available to the public that are based only on informal observations or anecdotes [35]. Our data should help to inform and augment existing lists of bee–attractive plants in addition to encouraging the use of sustainable, bee–attractive woody landscape plants to conserve and restore resources for urban pollinators.
